# Supplementary material for: Dynamics of DNA damage-induced nuclear inclusions are regulated by SUMOylation of Btn2
Source: Nat Commun. 2024 Apr 13;15:3215. doi: 10.1038/s41467-024-47615-8 (PMC11016081; doi:10.1038/s41467-024-47615-8)
Supplement: Supplementary file 1 — Supplementary Information [file 41467_2024_47615_MOESM1_ESM.pdf]

Supplementary Information

Dynamics of DNA damage-induced nuclear inclusions are regulated by SUMOylation of Btn2

Arun Kumar[1,2], Veena Mathew[1] and Peter C. Stirling[1,2,3]

[1] Terry Fox Laboratory, BC Cancer, 675 West 10th Avenue Vancouver BC, V5Z1L3.
[2] Department of Medical Genetics, University of British Columbia, Vancouver, BC
[3] Correspondence to P.C.S.: pstirling@bccrc.ca

Supplementary Information included:

Supplementary Figures 1-5

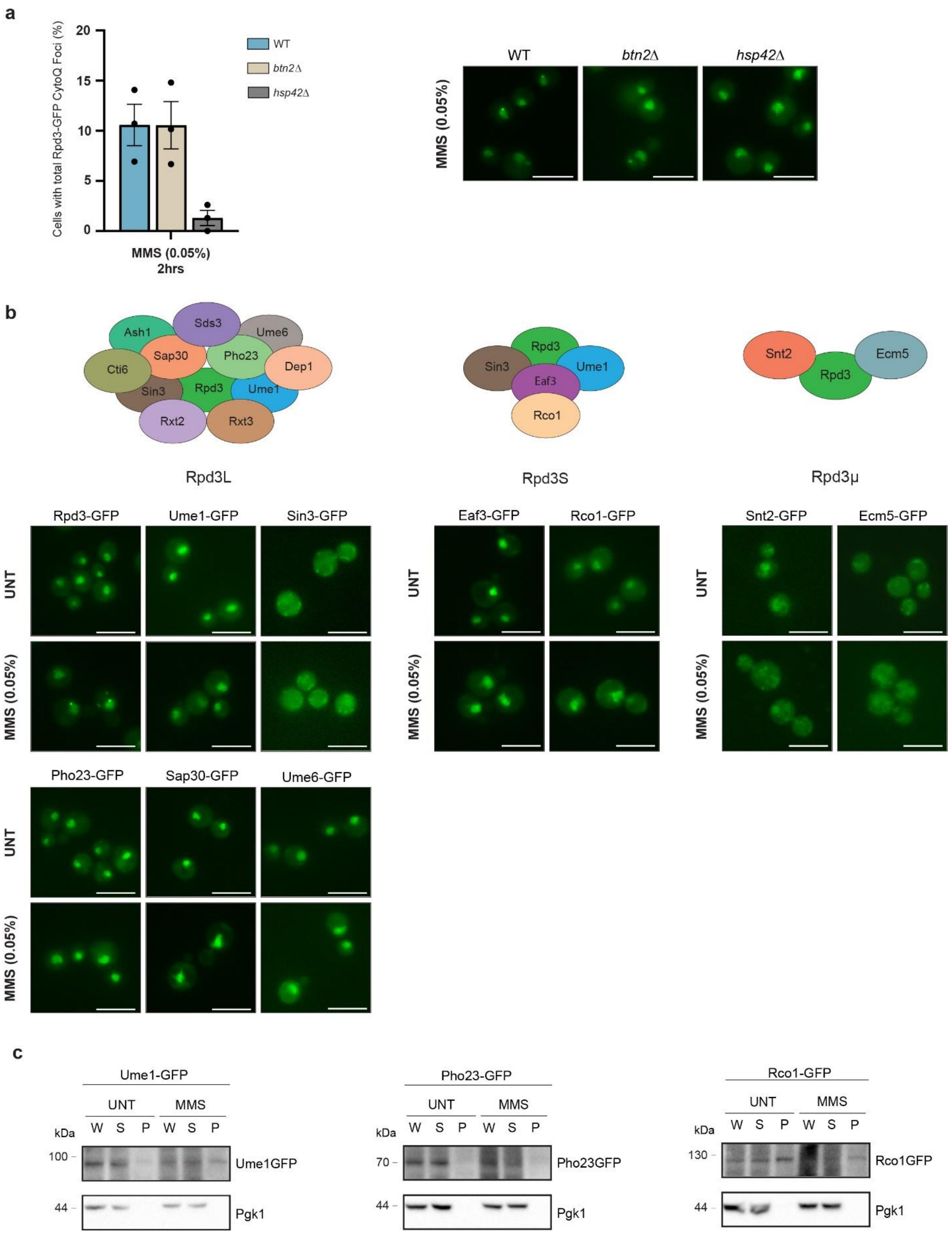
a
Cells with total Rpd3-GFP CytoQ Foci (%)
20
15
10
5
0
WT
btn2Δ
hsp42Δ
MMS (0.05%)
2hrs
WT
btn2Δ
hsp42Δ
MMS (0.05%)
b
Ash1
Sds3
Ume6
Sap30
Pho23
Dep1
Cti6
Sin3
Rpd3
Ume1
Rxt2
Rxt3
Rpd3
Sin3
Ume1
Eaf3
Rco1
Snt2
Ecm5
Rpd3
Rpd3L
Rpd3S
Rpd3μ
Rpd3-GFP
Ume1-GFP
Sin3-GFP
Eaf3-GFP
Rco1-GFP
Snt2-GFP
Ecm5-GFP
UNT
MMS (0.05%)
Pho23-GFP
Sap30-GFP
Ume6-GFP
c
Ume1-GFP
Pho23-GFP
Rco1-GFP
UNT
MMS
W S P W S P
kDa
100
44
70
130
Ume1GFP
Pho23GFP
Rco1GFP
Pgk1

Supplementary Figure 1. Rpd3 complex subunits are not sequestered to INQ under replication stress.
a Quantification of Rpd3-GFP CytoQ foci in WT, *btn2*Δ and *hsp42*Δ cells. Representative images are shown on the right. **b** Schematic of the various Rpd3-containing complexes and representative images of GFP-tagged Rpd3 complex subunits after MMS treatment. **c** Fractionation of Rpd3 complex subunits in both untreated and MMS-treated conditions followed by GFP western blots or an anti-Pgk1 loading control. UNT = untreated. W = whole cell extract; S = supernatant; P = pellet. All error bars represent means ± SEM, n=3 biologically independent replicates, >100 cells each. ****, p<0.0001, Fisher's test. Scale bars: 5µm.

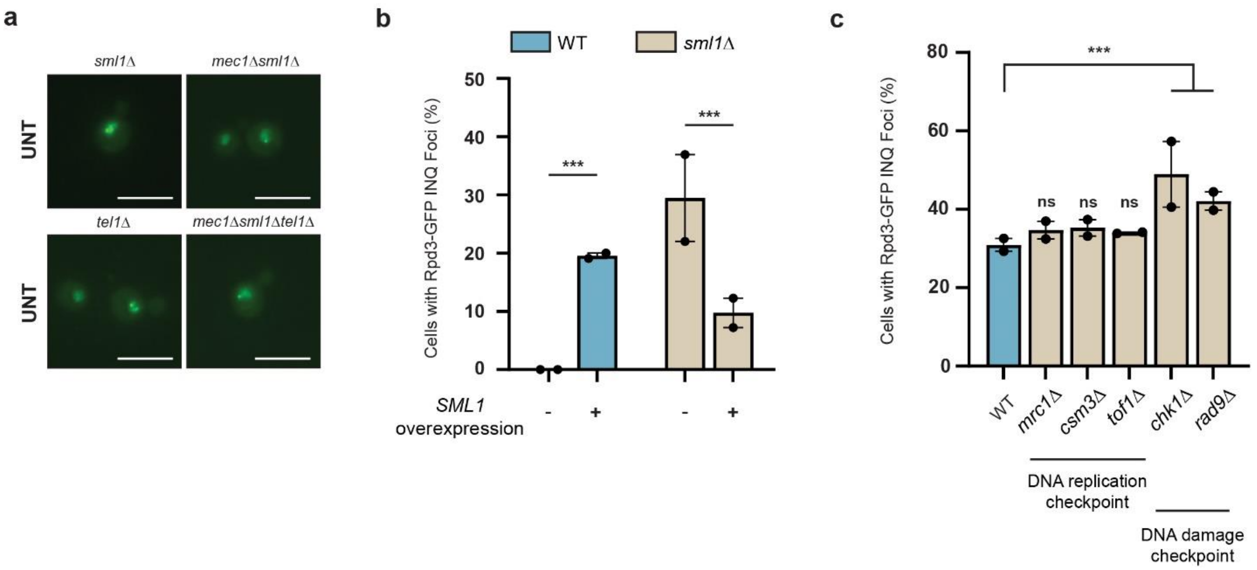

Supplementary Figure 2. Sequestration of Rpd3 to INQ in DNA damage response deficient backgrounds.
a Representative images for Rpd3 foci formation in untreated *sml1*Δ, *mec1*Δ*sml1*Δ, *tel1*Δ, and *mec1*Δ*sml1*Δ*tel1*Δ cells. **b** Rpd3-foci quantification showing Sml1 overexpression rescues *sml1*Δ INQ phenotype but also induces INQ formation in untreated cells. **c** Quantification of Rpd3 INQ formation in DNA repair deficient mutants. All error bars represent means ± SEM, n=2 biologically independent replicates, >100 cells each. ****, p<0.0001, ***, p<0.0002,ns, p>0.1, Fisher's test. Scale bars: 5µm.

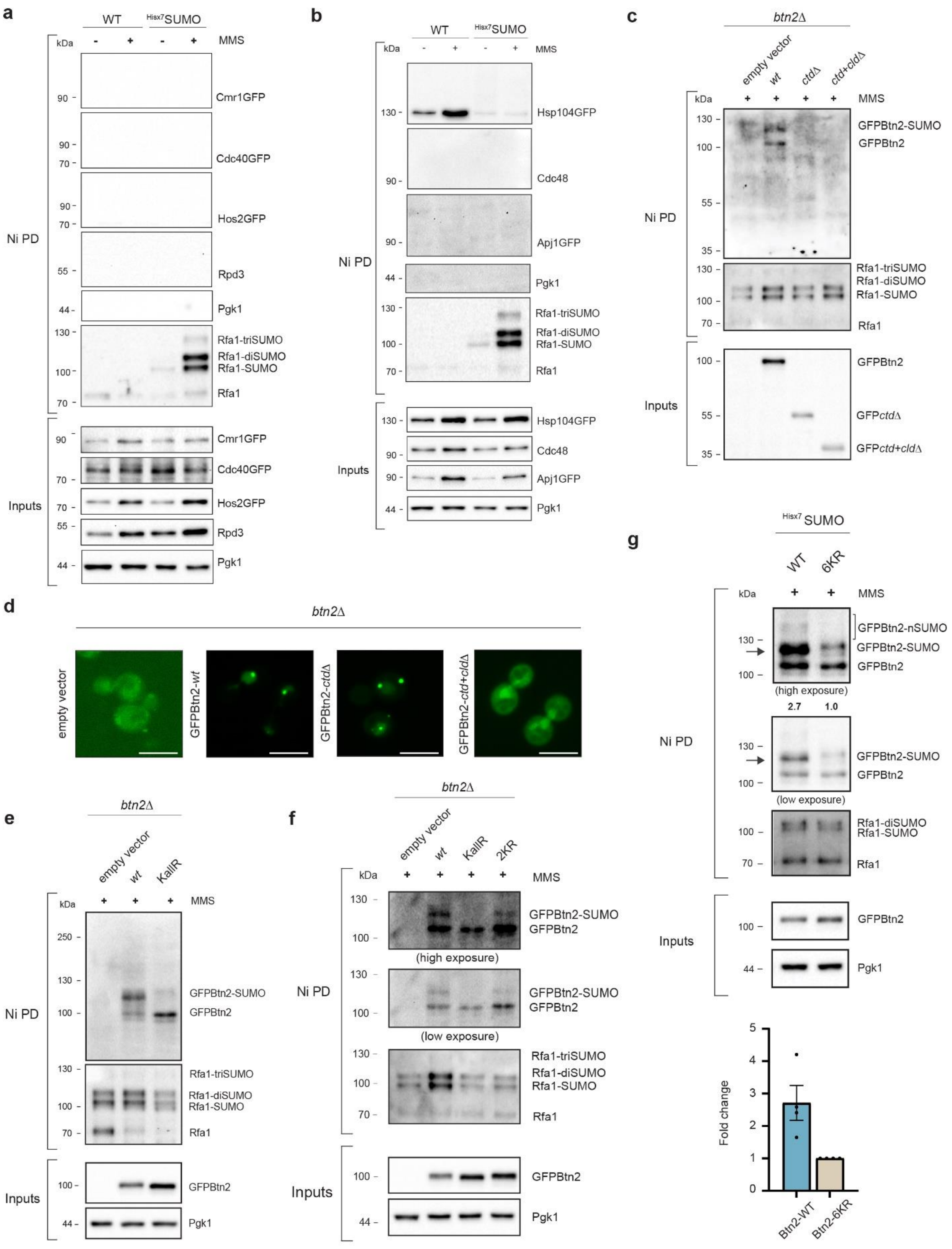
a
WT
Hisx7SUMO
MMS
kDa
Ni PD
Inputs
Cmr1GFP
Cdc40GFP
Hos2GFP
Rpd3
Pgk1
Rfa1-triSUMO
Rfa1-diSUMO
Rfa1-SUMO
Rfa1
b
Hsp104GFP
Cdc48
Apj1GFP
c
btn2Δ
empty vector
wt
ctdΔ
ctd+cldΔ
GFPBtn2-SUMO
GFPBtn2
GFPctdΔ
GFPctd+cldΔ
d
GFPBtn2-wt
GFPBtn2-ctdΔ
GFPBtn2-ctd+cldΔ
e
KallR
f
2KR
(high exposure)
(low exposure)
g
6KR
GFPBtn2-nSUMO
2.7
1.0
Fold change
Btn2-WT
Btn2-6KR

Supplementary Figure 3. Nickel-His Pulldowns for various INQ substrates and Btn2 mutants.
a, b SUMOylation-specific denaturing nickel bead pulldowns (NiPDs) of INQ substrates and chaperones respectively, as listed in **Fig. 4a.** **c** Second replicate of NiPDs for confirming the SUMOylation domain of Btn2. **d** Localization of GFP-Btn2 domain mutants in *btn2*Δ cells. **e** NiPD of GFP-Btn2 WT and the CTD-KallR constructs confirms CTD as the SUMOylated domain. **f** NiPDs of Btn2 CTD construct Btn2-2KR represented in **Fig. 4g**. **g** Genome integrated Btn2-6KR does not get SUMOylated upon DNA damage. Relative fold change was measured from four independent experiments. Scale bars: 5µm.

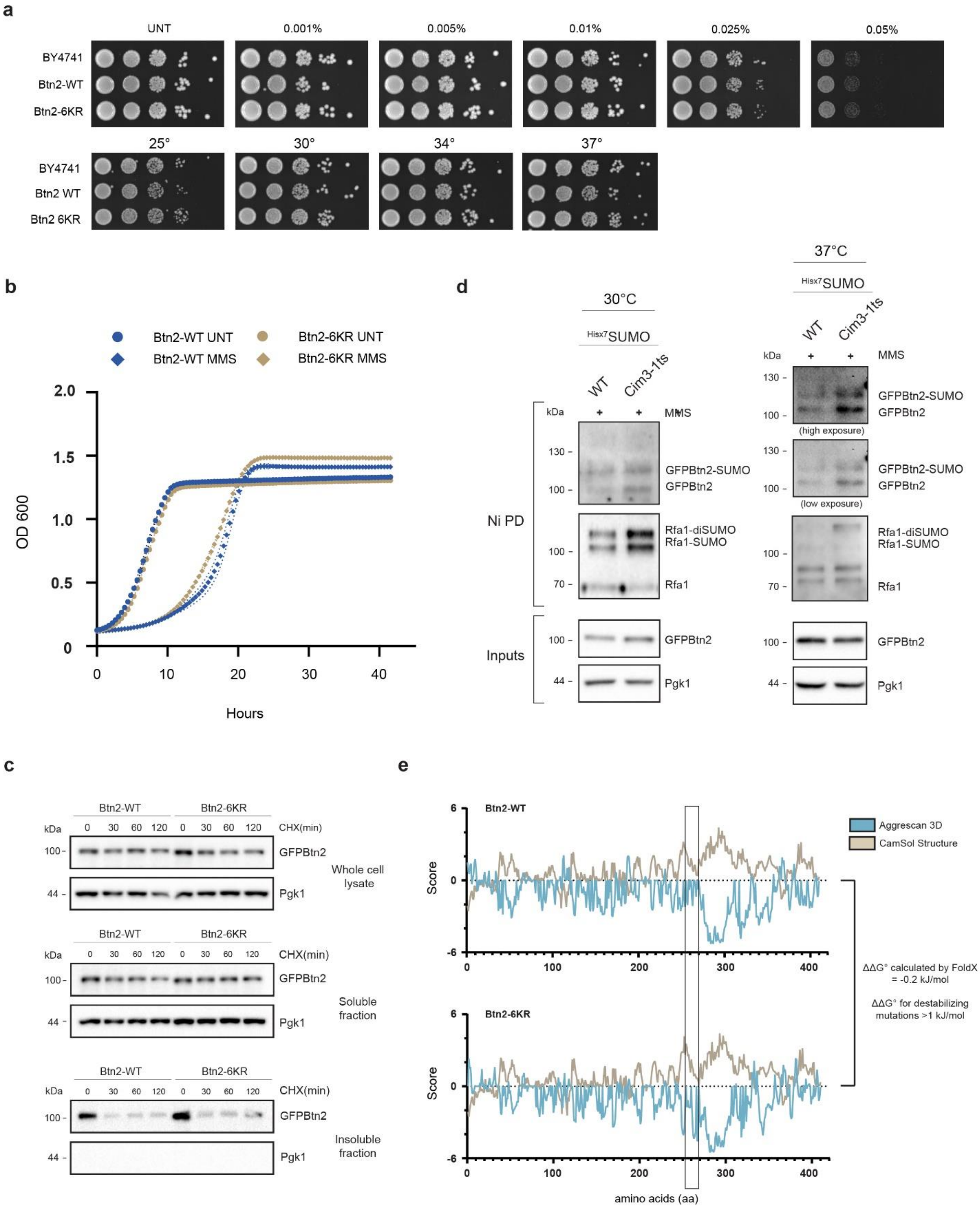

a
UNT
0.001%
0.005%
0.01%
0.025%
0.05%
BY4741
Btn2-WT
Btn2-6KR
25°
30°
34°
37°
BY4741
Btn2 WT
Btn2 6KR
b
Btn2-WT UNT
Btn2-WT MMS
Btn2-6KR UNT
Btn2-6KR MMS
OD 600
Hours
c
Btn2-WT
Btn2-6KR
kDa
CHX(min)
GFPBtn2
Pgk1
Whole cell lysate
Soluble fraction
Insoluble fraction
d
30°C
37°C
Hisx7SUMO
WT
Cim3-1ts
MMS
Ni PD
Inputs
GFPBtn2-SUMO
GFPBtn2
(high exposure)
(low exposure)
Rfa1-diSUMO
Rfa1-SUMO
Rfa1
Pgk1
e
Btn2-WT
Btn2-6KR
Aggrescan 3D
CamSol Structure
Score
amino acids (aa)
ΔΔG° calculated by FoldX = -0.2 kJ/mol
ΔΔG° for destabilizing mutations >1 kJ/mol

Supplementary Figure 4. Characterization of Btn2-6KR in comparison to Btn2-WT.

a Spot dilution assay comparing Btn2-WT and Btn2-6KR growth at 25°C, 30°C, 34°C, and 37°C and under MMS concentrations of 0.001%, 0.005%, 0.01%, 0.025% and 0.05% MMS. Images were taken after two days of spotting. **b** Growth curves analysis of Btn2-WT and Btn2-6KR cells after 2 h of MMS treatment followed by washout into MMS-free media compared with untreated cells. Dots surrounding data points indicate standard error of mean from three different replicates. **c** GFP-Btn2 stability and solubility with or without the 6KR mutation following cycloheximide chase. Pulse chase experiments were performed after treating cells with cycloheximide to halt translation and collected at various time points. **d** NiPD of GFP-Btn2 cells in WT vs proteasome defective *cim3-1 ts* allele at two non-permissive temperatures of 30°C and 37°C. All cells were treated with MMS. **e** CamSol and Aggrescan3D plots for Btn2-WT and Btn2-6KR. Graphs were plotted using scores provided by the respective softwares. Rectangular bin represents the Btn2-6KR mutation region.

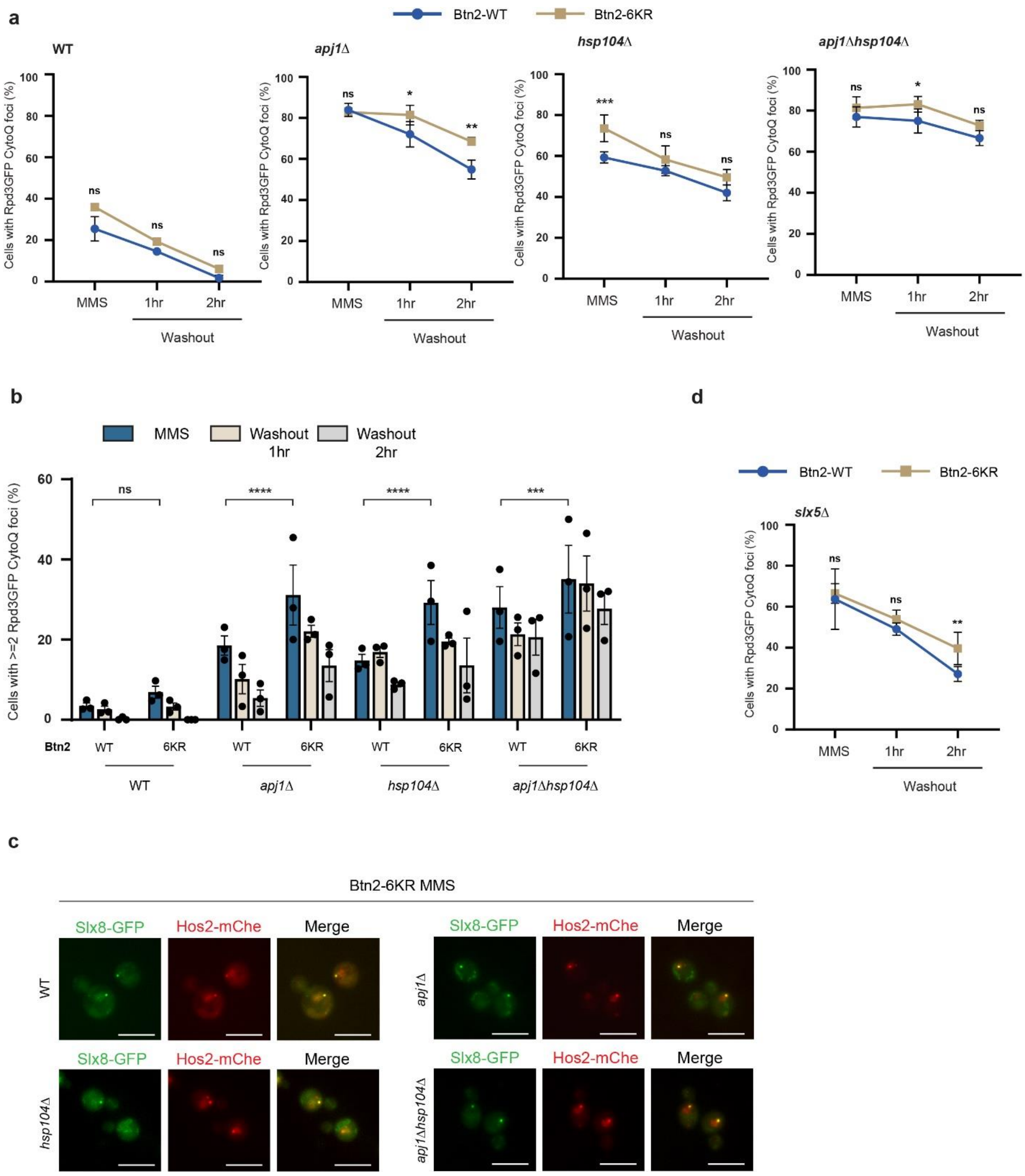

Supplementary Figure 5. Effect of Btn2 SUMOylation in cytoplasmic foci formation and clearance. **a** Quantification of peripheral CytoQ foci in INQ clearance-deficient washout experiments (related to Figure 6a). **b** Quantification of cells with greater than 1 peripheral aggregate in the strains from a. **c** Representative images for quantification of Slx8-GFP INQ foci from **Fig 6e** in different genetic backgrounds. **d** Washout of peripheral CytoQ Rpd3-GFP foci following MMS induction in cells lacking *SLX5* with or without Btn2-6KR. All error bars represent ± SEM, n=3, >100 cells each. ****, p<0.0001, ***, p<0.0002, **, p<0.002, *, p<0.03, ns, p>0.1 , Fisher's test.
