# Supplementary material for: Dynamics of DNA damage-induced nuclear inclusions are regulated by SUMOylation of Btn2
Source: Nat Commun. 2024 Apr 13;15:3215. doi: 10.1038/s41467-024-47615-8 (PMC11016081; doi:10.1038/s41467-024-47615-8)
Supplement: Supplementary file 3 — Description of Additional Supplementary Files [file 41467_2024_47615_MOESM3_ESM.pdf]

Description of Additional Supplementary Files

File Name: Supplementary Data 1

Description List of yeast strains, primers, plasmids, antibodies and statistical tests used in this study

Sheet 1 – Yeast strains

Sheet 2 – Primers

Sheet 3 – Plasmids

Sheet 4 – Antibodies

Sheet 5 – Statistical tests and values
