# Supplementary material for: Dynamics of DNA damage-induced nuclear inclusions are regulated by SUMOylation of Btn2
Source: Nat Commun. 2024 Apr 13;15:3215. doi: 10.1038/s41467-024-47615-8 (PMC11016081; doi:10.1038/s41467-024-47615-8)
Supplement: Supplementary file 5 — Reporting Summary [file 41467_2024_47615_MOESM5_ESM.pdf]

nature portfolio

Corresponding author(s): Peter C. Stirling

Last updated by author(s): Mar 8, 2024

Reporting Summary

Nature Portfolio wishes to improve the reproducibility of the work that we publish. This form provides structure for consistency and transparency in reporting. For further information on Nature Portfolio policies, see our Editorial Policies and the Editorial Policy Checklist.

Statistics

For all statistical analyses, confirm that the following items are present in the figure legend, table legend, main text, or Methods section.

n/a	Confirmed	
☐	☒	The exact sample size (*n*) for each experimental group/condition, given as a discrete number and unit of measurement
☐	☒	A statement on whether measurements were taken from distinct samples or whether the same sample was measured repeatedly
☐	☒	The statistical test(s) used AND whether they are one- or two-sided<br>*Only common tests should be described solely by name; describe more complex techniques in the Methods section.*
☒	☐	A description of all covariates tested
☐	☒	A description of any assumptions or corrections, such as tests of normality and adjustment for multiple comparisons
☐	☒	A full description of the statistical parameters including central tendency (e.g. means) or other basic estimates (e.g. regression coefficient) AND variation (e.g. standard deviation) or associated estimates of uncertainty (e.g. confidence intervals)
☐	☒	For null hypothesis testing, the test statistic (e.g. *F*, *t*, *r*) with confidence intervals, effect sizes, degrees of freedom and *P* value noted<br>*Give P values as exact values whenever suitable.*
☒	☐	For Bayesian analysis, information on the choice of priors and Markov chain Monte Carlo settings
☒	☐	For hierarchical and complex designs, identification of the appropriate level for tests and full reporting of outcomes
☒	☐	Estimates of effect sizes (e.g. Cohen's *d*, Pearson's *r*), indicating how they were calculated

Our web collection on statistics for biologists contains articles on many of the points above.

Software and code

Policy information about availability of computer code

Data collection	Data collection for microscopy was performed using MetaMorph Premier Acquisition Version 7.8.
Data analysis	Data analysis was carried out in GraphPad Prism 8.0 or higher, ImageLab 5.2.1 and ImageJ Version 5.3t.

For manuscripts utilizing custom algorithms or software that are central to the research but not yet described in published literature, software must be made available to editors and reviewers. We strongly encourage code deposition in a community repository (e.g. GitHub). See the Nature Portfolio guidelines for submitting code & software for further information.

Data

Policy information about availability of data

All manuscripts must include a data availability statement. This statement should provide the following information, where applicable:

- Accession codes, unique identifiers, or web links for publicly available datasets
- A description of any restrictions on data availability
- For clinical datasets or third party data, please ensure that the statement adheres to our policy

The published article includes all datasets generated or analyzed during this study. This includes the quantification of foci, western blots from Fig. 1f, Fig. 3d, Fig. 4b,c,e,g, Fig. 5b,e, Fig. 6c,d,f, Supp Fig. 1c, Supp Fig 3a-c,e-g, Supp Fig. 4c,d, Supp Fig. 5c and fractionation intensities that are provided as a Source Data file. Further information and requests for resources and reagents should be directed to and will be fulfilled by the authors upon reasonable request.

Research involving human participants, their data, or biological material

Policy information about studies with human participants or human data. See also policy information about sex, gender (identity/presentation), and sexual orientation and race, ethnicity and racism.

Reporting on sex and gender	N/A
Reporting on race, ethnicity, or other socially relevant groupings	N/A
Population characteristics	N/A
Recruitment	N/A
Ethics oversight	N/A

Note that full information on the approval of the study protocol must also be provided in the manuscript.

Field-specific reporting

Please select the one below that is the best fit for your research. If you are not sure, read the appropriate sections before making your selection.

☒ Life sciences ☐ Behavioural & social sciences ☐ Ecological, evolutionary & environmental sciences

For a reference copy of the document with all sections, see nature.com/documents/nr-reporting-summary-flat.pdf

Life sciences study design

All studies must disclose on these points even when the disclosure is negative.

Sample size	Sample size was not predetermined using any sample-size calculation. However, three individual biological replicates were performed for each experiment and genotype (unless otherwise specified in the figure legend), each performed on a different day and from a different starter culture. A sample size of three is an accepted standard in the field to answer and attain reproducible results for the questions asked in this study. Furthermore, ensuring independent biological replicates should account for variation between samples.
Data exclusions	No data was excluded.
Replication	All experiments were replicated using three independent cultures performed on three different days. See the methods section of each experiment for more detail.
Randomization	Samples were not allocated into experimental groups.
Blinding	Investigators were not blinded to sample identity since this study deals with isogenic strains of yeast where each question pertaining to genetic deletions were predetermined and not subjective making blinding of samples not relevant.

Reporting for specific materials, systems and methods

We require information from authors about some types of materials, experimental systems and methods used in many studies. Here, indicate whether each material, system or method listed is relevant to your study. If you are not sure if a list item applies to your research, read the appropriate section before selecting a response.

Materials & experimental systems

n/a	Involved in the study	
☐	☒	Antibodies
☒	☐	Eukaryotic cell lines
☒	☐	Palaeontology and archaeology
☐	☒	Animals and other organisms
☒	☐	Clinical data
☒	☐	Dual use research of concern
☒	☐	Plants

Methods

n/a	Involved in the study	
☒	☐	ChIP-seq
☒	☐	Flow cytometry
☒	☐	MRI-based neuroimaging

Antibodies

Antibodies used	Mouse anti-Pgk1 (sc-130335) (Santa Cruz Biotechnology),<br>Mouse anti-GFP (B-2) (sc-9996) (Santa Cruz Biotechnology),<br>Mouse anti-Ubiquitin (P4D1) (3936s) (Cell Signaling Technology),<br>Rabbit anti-K48-linkage Specific Polyubiquitin (D9D5) (8081S) (Cell Signaling Technology),<br>Rabbit anti-Smt3 (ab14405) (Abcam),<br>Rabbit anti-Rfa (AS07214) (Agrisera),<br>Mouse anti-Rpd3 (sc-514160) (Santa Cruz Biotechnology),<br>Mouse anti-p97/VCP/Cdc48 (NB120-11433) (Novus Biologicals)
Validation	All primary antibodies mentioned have been validated by their manufacturers.<br>anti-Pgk1 (1:500): see manufacturer's web page for 33 product citations and Tam, AS. et al. 2019 Mol Biol Cell. and Mathew, V. et al. 2020 J Cell Sci.<br>anti-GFP (1:500): see manufacturer's web page for 3295 product citations.<br>anti-Ubiquitin (1:1000): see manufacturer's web page for 932 product citations.<br>anti-K48-linkage specific polyubiquitin (1:1000): see manufacturer's web page for 202 citations along with validation by T. Mayor's group in Fang, NN. et al. 2016 Nat Commun.<br>anti-Smt3 (1:1000): see manufacturer's web page for 9 citations.<br>anti-Rfa (1:1000): see manufacturer's web page for citations including Mathew, V. et al. 2020 J Cell Sci.<br>anti-Rpd3 (1:500): see manufacturer's web page for 2 citations.<br>anti-Cdc48 (1:1000): see manufacturer's web page for 3 citations.

Animals and other research organisms

Policy information about studies involving animals; ARRIVE guidelines recommended for reporting animal research, and Sex and Gender in Research

Laboratory animals	N/A
Wild animals	N/A
Reporting on sex	Not applicable as yeast was used the model organism for the study. The S. cerevisiae strains used were S288C isogenic strains MATa BY4741 unless otherwise mentioned in Supplementary Data 1.
Field-collected samples	N/A
Ethics oversight	N/A

Note that full information on the approval of the study protocol must also be provided in the manuscript.
